# Supplementary figures and images for: Cold dispase digestion of murine lungs improves recovery and culture of airway epithelial cells
Source: PLoS One. 2024 Jan 25;19(1):e0297585. doi: 10.1371/journal.pone.0297585 (PMC10810513; doi:10.1371/journal.pone.0297585)

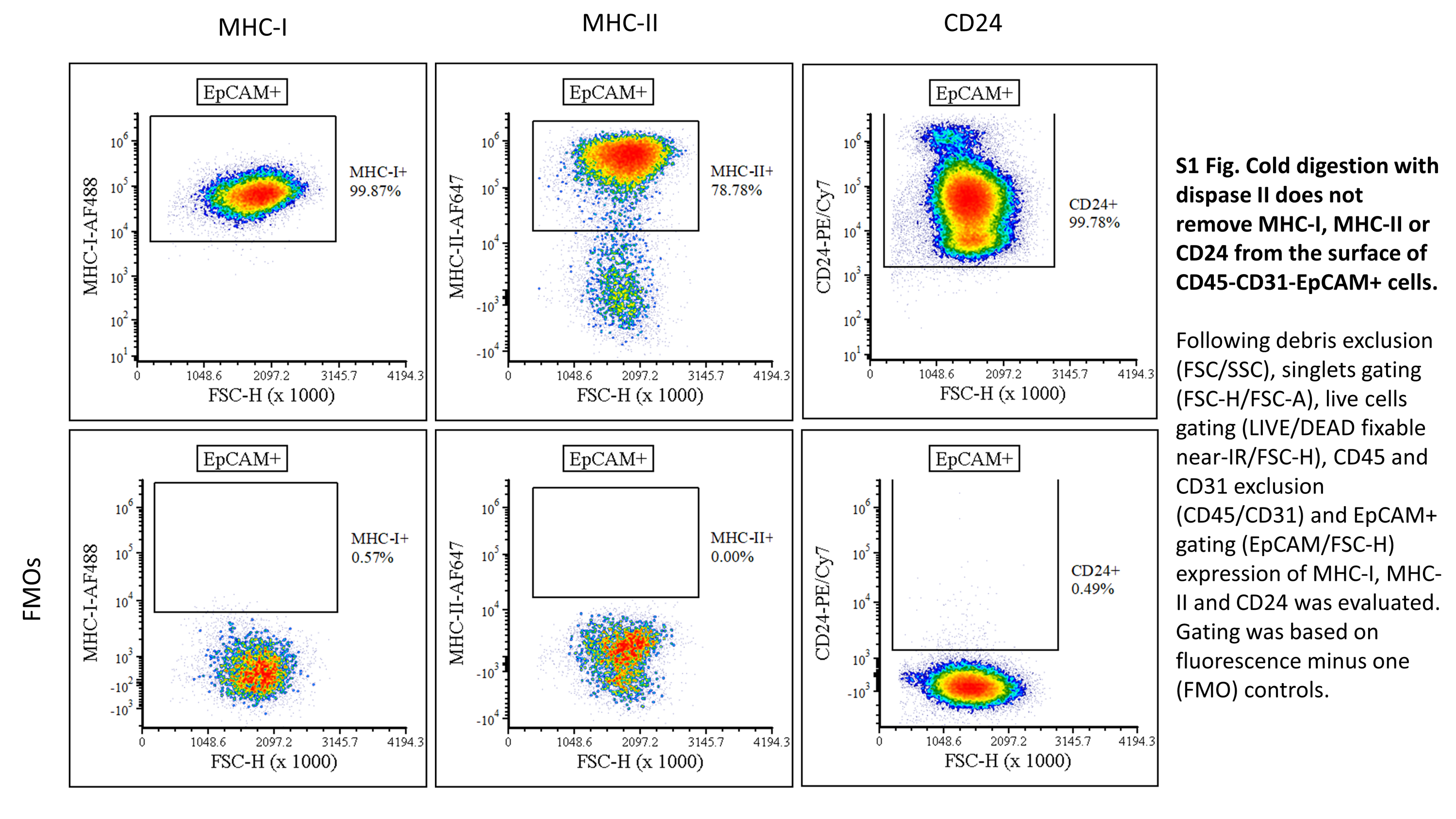

Supplement: S1 Fig — Following debris exclusion (FSC/SSC), singlets gating (FSC-H/FSC-A), live cells gating (LIVE/DEAD fixable near-IR/FSC-H), CD45 and CD31 exclusion (CD45/CD31) and EpCAM+ gating (EpCAM/FSC-H) expression of MHC-I, MHC-II and CD24 was evaluated. Gating was based on fluorescence minus one (FMO) controls. (TIF) [file pone.0297585.s001.tif]

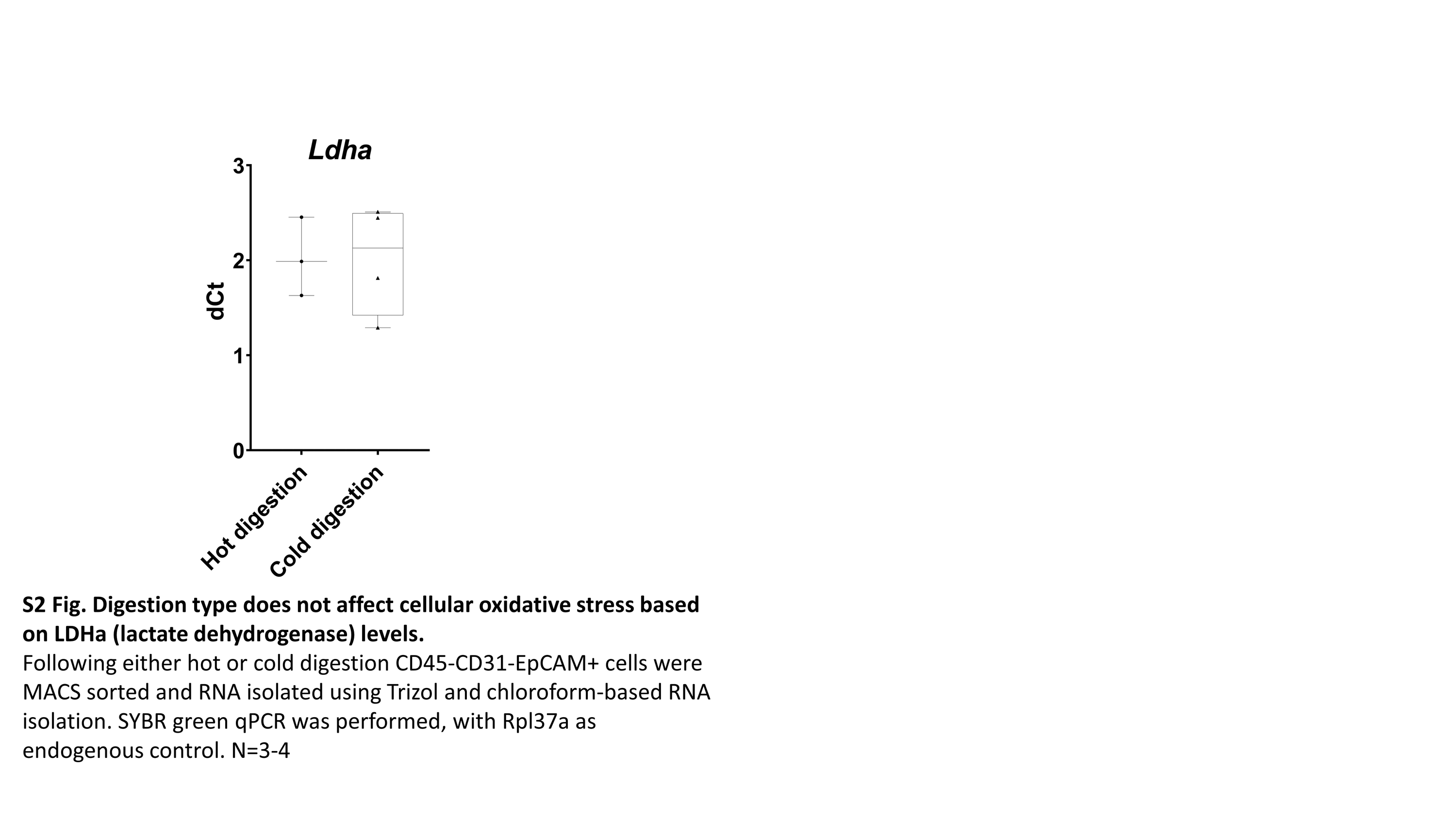

Supplement: S2 Fig — Following either hot or cold digestion CD45-CD31-EpCAM+ cells were MACS sorted and RNA isolated using Trizol and chloroform-based RNA isolation. SYBR green qPCR was performed, with Rpl37a as endogenous control. N = 3–4. (TIF) [file pone.0297585.s002.tif]

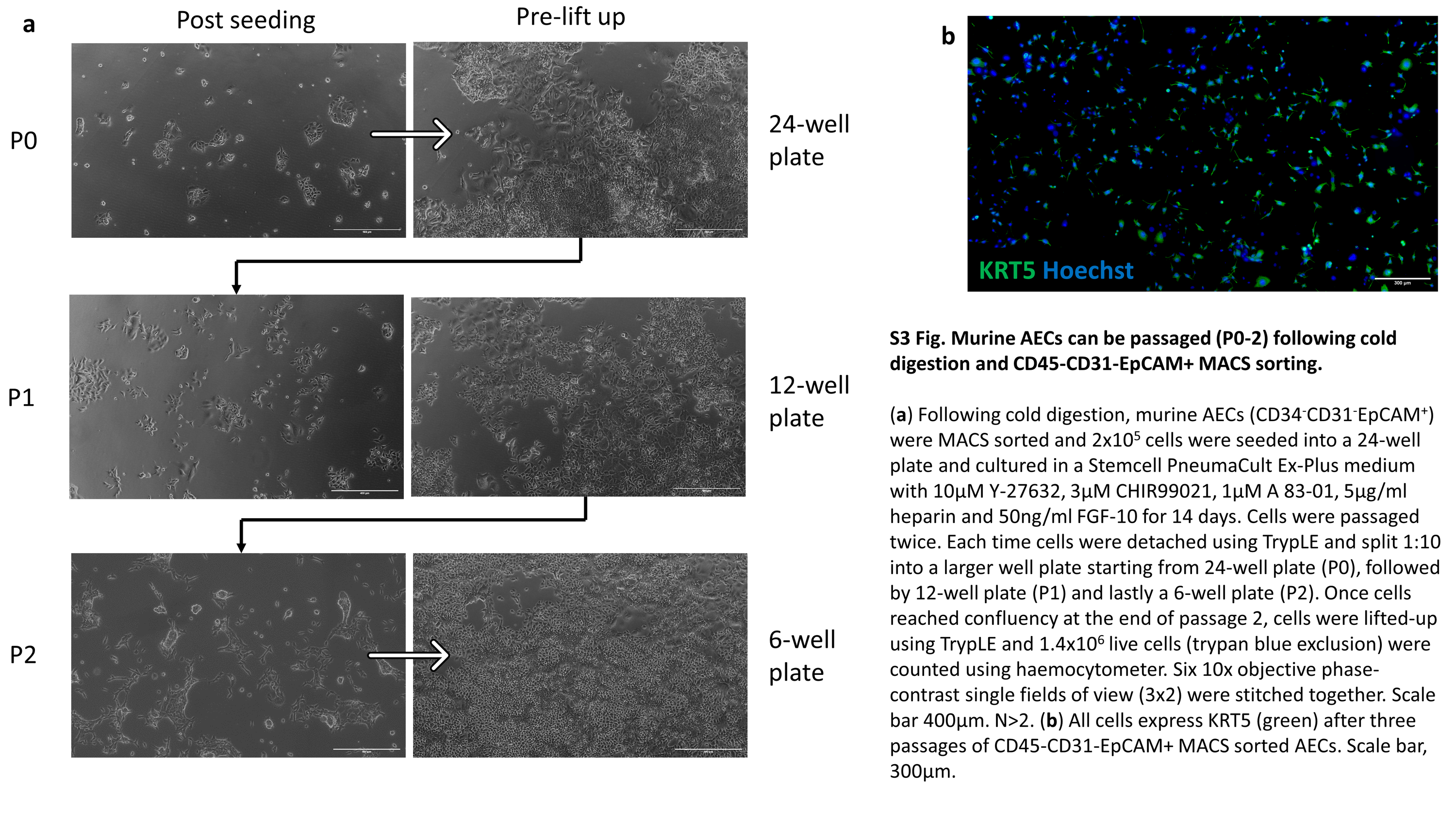

Supplement: S3 Fig — (a) Following cold digestion, murine AECs (CD34-CD31-EpCAM+) were MACS sorted and 2x10^5^ cells were seeded into a 24-well plate and cultured in a Stemcell PneumaCult Ex-Plus medium with 10μM Y-27632, 3μM CHIR99021, 1μM A 83–01 for 14 days. Cells were passaged twice. Each time cells were detached using TrypLE and split 1:10 into a larger well plate starting from 24-well plate (P0), followed by 12-well plate (P1) and lastly a 6-well plate (P2). Once cells reached confluency at the end of passage 2, cells were lifted up using TrypLE and 1.4x10^6^ live cells (trypan blue exclusion) were counted using haemocytometer. Six 10x objective phase-contrast single fields of view (3x2) were stitched together. Scale bar 400μm. N>2. (b) All cells express KRT5 (green) after three passages of CD45-CD31-EpCAM+ MACS sorted AECs. Scale bar, 300μm. (TIF) [file pone.0297585.s003.tif]
