# Supplementary material for: Cold dispase digestion of murine lungs improves recovery and culture of airway epithelial cells
Source: PLoS One. 2024 Jan 25;19(1):e0297585. doi: 10.1371/journal.pone.0297585 (PMC10810513; doi:10.1371/journal.pone.0297585)
Supplement: S1 File — https://dx.doi.org/10.17504/protocols.io.rm7vzxo68gx1/v1. (PDF) [file pone.0297585.s005.pdf]

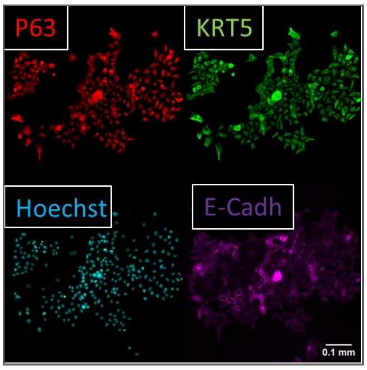

OCT 29, 2023

OPEN ACCESS

DOI:
dx.doi.org/10.17504/protocols.io.rm7vzxo68gx1/v1

Protocol Citation: piotr.janas, Caroline Chauché, Patrick Shearer, Georgia Perona-Wright, Henry J McSorley, Jürgen Schwarze 2023. Cold dispase digestion of murine lungs improves recovery and culture of airway epithelial cells. **protocols.io** https://dx.doi.org/10.17504/protocols.io.rm7vzxo68gx1/v1

License: This is an open access protocol distributed under the terms of the Creative Commons Attribution License, which permits unrestricted use, distribution, and reproduction in any medium, provided the original author and source are credited

Protocol status: Working
We use this protocol and it's working

Cold dispase digestion of murine lungs improves recovery and culture of airway epithelial cells

piotr.janas[1], Caroline Chauché[2], Patrick Shearer[3], Georgia Perona-Wright[3], Henry J McSorley[4], Jürgen Schwarze[1]

[1]The University of Edinburgh; [2]The Univeresity of Edinburgh; [3]The University of Glasgow; [4]University of Dundee

piotr.janas

ABSTRACT

Airway epithelial cells (AECs) play a key role in maintaining lung homeostasis, epithelium regeneration and the initiation of pulmonary immune responses. To isolate and study murine AECs investigators have classically used short and hot (1h 37°C) digestion protocols. Here, we present a workflow for efficient AECs isolation and culture, utilizing long and cold (20h 4°C) dispase II digestion of murine lungs. This protocol yields a greater number of viable AECs compared to an established 1h 37°C dispase II digestion. Using a combination of flow cytometry and immunofluorescent microscopy, we demonstrate that compared to the established method, the cold digestion allows for recovery of a 3-fold higher number of CD45-CD31-EpCAM+ cells from murine lungs. Their viability is increased compared to established protocols, they can be isolated in larger numbers by magnetic-activated cell sorting (MACS), and they result in greater numbers of distal airway stem cell (DASC) KRT5+p63+ colonies in vitro. Our findings demonstrate that temperature and duration of murine lung enzymatic digestion have a considerable impact on AEC yield, viability, and ability to form colonies in vitro. We believe this workflow will be helpful for studying lung AECs and their role in the biology of lung.

MATERIALS

• Dissection scissors
• Blunt curved dissection tweezers
• Spring bow scissors
• Elastic string
• Blunt G20 1” needle
• Dulbecco's Modified Eagle Medium: Nutrient Mixture F12 (DMEM/F12) (Gibco, 11320033)
• Penicillin-Streptomycin (Gibco, 15140122)
• Dulbecco’s phosphate-buffered saline (DPBS) (Gibco, 14190144)
• 24-well plate (Costar, 3524)
• Collagen (Sigma-Aldrich, C8919-20ml)
• Fibronectin (Merck, F2006-1MG)

Created: Oct 29, 2023

Last Modified: Oct 29, 2023

PROTOCOL integer ID: 90074

Keywords: DASC, respiratory epithelial cells, lung epithelial cells, LECs, AECs, airway epithelial cells, MACS, cell isolation, dispase digestion, cold digestion

Funders Acknowledgement:
British Lung Fundation
Grant ID: PHD16-19 BUSH
Horserace Betting Levy Board
Grant ID: VET/2020 -1 EPDF 7

- Bovine Serum Albumin (BSA) (Sigma-Aldrich, A7030-500G)
- Hanks' Balanced Salt Solution (HBSS) (Gibco, 14025092)
- 70µm strainers (Miltenyi, 130-098-458)
- 40µm strainers (Miltenyi, 130-098-462)
- Dispase II (Sigma-Aldrich, D4693-1G)
- DNase I (Roche, 10104159001)
- 5ml Bijous
- 50ml falcon tubes
- ACK Lysing Buffer (Gibco, A1049201)
- Anti-CD45 MACS beads (Miltenyi, 130-052-301)
- Anti-CD31 MACS beads (Miltenyi, 130-097-418)
- Anti-EpCAM MACS beads (Miltenyi, 130-105-958)
- LS MACS columns (Miltenyi, 130-042-401)
- MS MACS columns (Miltenyi, 130-042-201)
- Fc block CD16/CD32 (BioLegend, 101301)
- OctoMACS separator (Miltenyi, 130-042-109)
- QuadroMACS separator (Miltenyi, 130-091-051)
- EDTA (Gibco, 15575020)
- Airway Epithelial Cell Growth Medium (Promocell, C-21060)
- DMH-1 (StemCell, 73632)
- A 83-01 (StemCell, 72022)
- Y-27632 (StemCell, 72302)
- CHIR9901 (StemCell, 72052)
- Trypan Blue (Gibco, 15250061)
- Stericup filter unit (Milipore, S2GPT01RE)
- 5ml syringes
- Haemocytometer

Recipes

MACS buffer wash - DPBS (no Mg^{2+} and Ca^{2+}) + 0.5% BSA + 2mM EDTA + 1% Pen/Strep

Well-plate coating solution - 3% collagen, 1% fibronectin and 1% BSA in HBSS. Distribute at least 0.2ml per $1cm^2$. Incubate for at least 4-8h at 37°C, then keep up to 5 weeks at 4C⁰ covered in film.

Airway epithelial growth media – 1µM A 83-01, 0.2µM DMH-1, 5µM Y27632 ROCK, 0.5µM CHIR9901 and 1% v/v Penicillin/Streptomycin in supplemented Promocell Airway Epithelial Cell Growth Medium. Keep up to 7 days at 4°C and protected from light.

Digestion solution - 2mg/ml of Dispase II resuspended in sterile DMEM/F12 +

0.1mg/ml DNase I + 1% v/v Penicillin/Streptomycin filtered through 0.22µm filter. Aliquot to Bijous (5ml) and freeze down. Thaw aliquots one hour before the start of the protocol.

Dispase wash - 50µg/ml of DNaseI with 1% v/v Penicillin/Streptomycin in DMEM/F12.

Cold dispase digestion of murine lungs improves recovery an...

1 Euthanise a mouse according to local protocols and regulations (excluding carbon dioxide).

2 Using scissors, cut the skin and open the abdomen. Follow through by cutting the skin along the sternum and neck.

3 Cut the abdominal aorta.

4 Expose the trachea by removing salivary glands. Support the trachea by releasing curved tweezers underneath the trachea.

5 Make a small incision below the larynx using spring bow scissors.

6 Cannulate the trachea using a blunt needle and secure it using elastic thread.

7 Perform bronchoalveolar lavage (BAL) three times with pre-warmed (37°C) DMEM/F12 (0.8ml/BAL/mouse).

8 Remove the ribcage and flush the cardiovascular system by injecting 10ml of ice-cold DMEM/F12 into the right ventricle.

9 Using the tracheal cannula inject 1.5-2ml of ice-cold digestion solution into the lungs.

10 Remove the lungs by dissecting them from thoracic cavity. Cut at the primary bronchial bifurcation and quickly place lungs in remaining ice-cold 3ml of digestion mixture before the lungs deflate.

11 Incubate the lungs for 20h at 4°C.

12 Pour the digestion solution together with the lungs onto a 70µm strainer and mash the lungs using 5ml syringe plunger by gently agitating the lungs on the mesh (lungs should dissolve without a need for excessive force).

13 Wash the strainer with 10ml of dispase wash and then centrifuge each sample for 15min, 4°C, 130 relative centrifugal force (RCF).

14 Remove the supernatant and add 2ml of ice-cold ACK lysing buffer (red blood cell lysis) to each tube. Swirl the samples for 90s and then add 5ml of MACS buffer.

15 Pass the samples through 40µm strainer, wash the strainer with 5ml of MACS buffer and centrifuged for 5min, 4°C at 300 RCF.

16 Resuspend each sample in 1ml of MACS buffer and block with 5µl of 0.5mg/ml anti-mouse CD16/32 antibody for at least 20min.

17 Count the number of cells using trypan blue and a haemocytometer.

18 After incubation, centrifuge cells for 5min, 4°C, 300 RCF and resuspend in 85µl of MACS buffer + 5µl of anti-CD31 microbeads and 10µl of anti-CD45 microbeads per 10^7 total cells.

19 Incubate the samples for 30 minutes on ice.

20 Wash each sample with 1ml/107 cells of MACS buffer and centrifuge for 5min, 4°C at 300 RCF.

21 Pass the samples through activated LS MACS columns on a QuadroMACS separator and collect the flow-through.

22 Centrifuge collected cells for 5min, 4°C, 300 RCF and remove supernatant.

23 Resuspended cells in left-over liquid

24 Add 15µl of anti-EpCAM microbeads and incubate samples on ice for 30 minutes.

25 Wash samples with 1ml MACS buffer, and centrifuge for 5min, 4°C, 300 RCF.

26 Discard supernatant and resuspend cells in 500µl of MACS buffer.

27 Pass the cell suspension through activated MS MACS columns using an OctoMACS separator.

28 Flush out EpCAM+ cells into a fresh test tube from the column by applying 2ml of MACS buffer and inserting the plunger into the column.

29 Centrifuge cells for 5min at 4°C, 300 RCF, remove supernatant, and resuspend cell pellet in 5ml of supplemented airway epithelial growth media.

30 Count the number of live cells using trypan blue and a haemocytometer.

31 Coat the well plate with well-plate coating solution and incubate for at least 4-8h at 37°C before use.

32 Wash the coated 24-well plate with DPBS before adding 0.4ml of pre-warmed airway epithelial growth medium.

33 Add at least $2x10^5$ live MACS sorted cells per well and change media after 24h, followed by media changes every two days thereafter. Within 7 days p63+ KRT5+ colonies will appear.
